# Effect of a Novel Transition Program on Disability After Stroke: A Trial Protocol

**DOI:** 10.1001/jamanetworkopen.2019.12356

**Published:** 2019-10-02

**Authors:** Emily Somerville, Brittany Minor, Marian Keglovits, Yan Yan, Susan Stark

**Affiliations:** 1 School of Medicine, Washington University in St Louis, St Louis, Missouri

## Abstract

**Importance:**

A gap in care for stroke survivors exists at the point of transition from inpatient rehabilitation to home, when survivors encounter new environmental barriers because of the cognitive and sensorimotor sequelae of stroke. Resolving these barriers and improving independence in the community have the potential to significantly improve stroke survivors’ long-term morbidity.

**Objective:**

To investigate the efficacy and safety of a novel enhanced rehabilitation transition program to reduce environmental barriers and improve daily activity performance and community participation among stroke survivors.

**Design, Setting, and Participants:**

This is a phase 2b, single-blind, parallel-group, randomized clinical trial. Participants will be randomized using a 1:1 allocation ratio, stratified by Functional Independence Measure and age, to either attentional control or the intervention. Community Participation Transition After Stroke (COMPASS) is a complex intervention that uses 2 complementary evidence-based interventions: home modifications and strategy training delivered in the home. Community participation after stroke, measured by the Reintegration to Normal Living Index, is the primary outcome. Secondary outcomes include quality of life after stroke, measured by the Stroke Impact Scale, and daily activity performance and magnitude of environmental barriers in the home, both measured by the In-Home Occupational Performance Evaluation. An intention-to-treat analysis will be used. A total of 180 participants, who are 50 years or older, were independent in activities of daily living prior to stroke, and are undergoing inpatient rehabilitation following stroke with a plan to be discharged home, will be included in the study.

**Discussion:**

Stroke is a leading cause of serious long-term disability in the United States. The COMPASS study is ongoing. To date, 99 participants have been recruited and 77 randomized, with 37 in the treatment group and 40 in the control group. Resumption of previous activities immediately after discharge can improve immediate and long-term community participation. Results from this study will fill a critical gap in stroke rehabilitation evidence by providing important information about the long-term community participation and daily activity performance among stroke survivors as well as environmental barriers in their homes.

**Trial Registration:**

ClinicalTrials.gov identifier: NCT03485820

## Key Points

**Question:**

Is a novel enhanced rehabilitation transition program, Community Participation Transition After Stroke (COMPASS), more effective at improving community participation and daily activity performance and reducing environmental barriers among stroke survivors than an equivalent dose of attentional control?

**Findings:**

In this phase 2b, single-blind, parallel-group, randomized clinical trial, 180 adults who have had ischemic or hemorrhagic strokes will be studied as they transition home from inpatient rehabilitation.

**Meaning:**

Removing environmental barriers faced by stroke survivors as they transition home may improve daily activity performance as well as home and community participation.

## Teaser

This trial protocol describes a randomized clinical trial that will investigate the efficacy and safety of a novel enhanced rehabilitation program to reduce environmental barriers and improve daily activity performance and community participation among stroke survivors.

## Introduction

Stroke is highly prevalent, costly, and disabling. Stroke is a leading cause of long-term disability in the United States.^1^ Half of stroke survivors are dependent on caregivers to perform their activities of daily living (ADLs).^2,3^ Unless a solution is identified to improve the long-term outcome of stroke survivors, annual costs attributed to stroke in the United States are projected to increase to $240.67 billion by 2030.

The transition from inpatient rehabilitation (IR) to home is an important window of opportunity for intervention.^4,5^ Resumption of previous activities immediately after discharge,^6^ at a time when people with stroke report struggling to reestablish daily routines,^5^ can improve immediate and long-term community reintegration. Providing environmental support improves performance of ADLs but is unproven among stroke survivors. Strategy training enables patients to identify and prioritize ADL problems, barriers to performance, and strategies to resolve the barriers.^7,8^ Community Participation Transition after Stroke (COMPASS) is a novel program that combines environmental modifications and strategy training during the transition from IR to home to facilitate community reintegration after stroke. If effective, this program will reduce disability in ADL performance and improve participation outcomes.

The primary objective of this study is to compare the efficacy of COMPASS with an equivalent dose of attentional control (AC; stroke education) for significant improvements in the primary outcome (community participation) and secondary outcomes (ADL performance and a reduction in environmental barriers in the home after stroke). Secondary objectives of this study include evaluating alternative outcome measures of participation, function, patient-reported quality of life, and caregiver burden that permit comparison with other stroke clinical trials and confirm the safety of COMPASS and evaluating process outcomes such as reach, cost, fidelity, and adherence.

## Methods

### Study Design

The study is a phase 2b randomized clinical trial that includes 180 patients receiving IR. We will compare COMPASS with an AC group for superiority. The Standard Protocol Items: Recommendations for Interventional Trials (SPIRIT) reporting guideline was followed in developing this protocol. The full trial protocol is available in the Supplement.

### Study Setting and Population

This study takes place in the homes of participants living within 60 miles of the St Louis, Missouri, metropolitan area. We are recruiting patients who have had an acute stroke, are 50 years or older, were independent in ADL performance prior to stroke, plan to discharge to home, and are medically stable. Our initial recruitment plan included only participants with ischemic stroke. We modified our inclusion criteria to include participants with a diagnosis of hemorrhagic stroke based on recommendations from reviewers and from physicians on the study team. We are excluding survivors with terminal diseases that limit life expectancy to less than 6 months, previously diagnosed cognitive disorders (eg, dementia) or cognitive impairment after stroke that makes interpretation of the self-rated scales difficult (ie, Short Blessed Test^9^ score of ≤10), moderate to severe aphasia (National Institutes of Health Stroke Scale^10^ Best Language rating of ≥2), or who reside in a congregate living facility. We are also recruiting the primary informal caregivers of enrolled participants. Caregivers must be 18 years or older and speak English to participate. Participant flow is outlined in the Figure.

**Figure.**
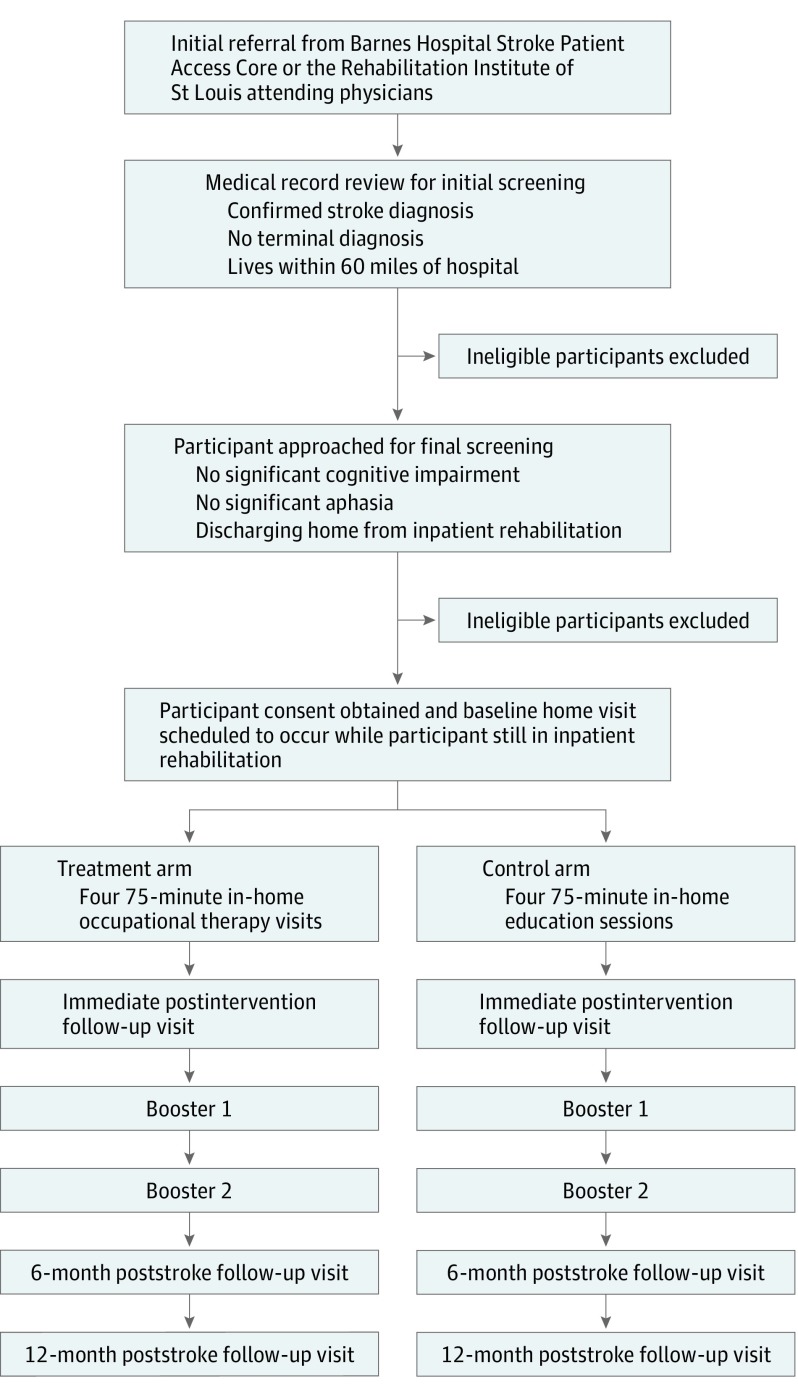
Flow Diagram of the Community Participation Transition After Stroke (COMPASS) Trial

### Recruitment and Consent

Participants are recruited near the time of transfer from acute care to IR and during IR. A study team member visits all patients who meet the inclusion criteria and invites them to participate in the study. Written informed consent is obtained. Caregivers provide consent during 1 of 4 treatment or control visits after the participant with stroke has returned home. All study procedures have been approved by the institutional review board at Washington University in St Louis.

### Randomization and Blinding

Participants are allocated into the education control group or the home modification intervention group using a 1:1 ratio via randomization sequences generated a priori by the study statistician using a computerized formal probability model. Functional status is a strong predictor of recovery.^11^ Therefore, randomization is balanced using the participant’s IR admission Functional Independence Measure^12^ score. There are 3 allocation strata for Functional Independence Measure scores; each strata corresponds with a level of functioning, ie, low, moderate, or high. Age is also a predictor of stroke outcomes, so randomization is balanced on age as well. There are 5 allocation strata for age, with each age block divided into 10-year increments, starting with age 50 years. There are a total of 15 strata, and we randomly allocate participants into 1 of 2 groups: treatment or control. Randomization sequence concealment will be achieved by query of the Research Electronic Data Capture (REDCap) system.^13^ After the baseline assessment (T1a), results are securely uploaded and stratification variables (ie, Functional Independence Measure score and age) are entered and locked. The interventionist completing the T1a elicits the treatment assignment in the field in real time using a secure data connection to REDCap, allowing in-home treatment of the participants assigned to the intervention group to begin immediately.

Inpatient rehabilitation staff from The Rehabilitation Institute of St Louis (TRISL) are blinded to allocation so that they do not modify their inpatient or discharge treatment plans. Follow-up raters are blind to allocation. To determine the effectiveness of our single-blinded protocol, we ask the rehabilitation therapists to complete a brief assessment to determine whether group assignments were revealed during evaluation. All incidents of unblinding are documented as protocol violations.

### Intervention

#### Study Procedures

The COMPASS manual fully defines and justifies each element of the intervention. The treatment includes 1 predischarge^14,15^ and four 75-minute postdischarge^16^ visits. The intervention is followed by 2 booster sessions.

#### Baseline Home Visit for All Participants With Stroke

Prior to randomization and discharge, an occupational therapy interventionist conducts a baseline activity assessment in the home (T1a). We use the In-Home Occupational Performance Evaluation (I-HOPE)^17^ to establish baseline activity patterns and identify environmental barriers in the home.

#### Telephone Assessment

Participation assessments (T1b) are conducted for both groups by telephone 2 days after discharge from IR to allow time for participants to adjust and personally assess their community participation. A blinded rater conducts the assessment for the primary, secondary, and exploratory end points.

#### Baseline Home Visit for Caregivers

If a caregiver is present, the occupational therapy interventionist or a trained graduate assistant collects basic demographic information from the caregiver and asks questions regarding stress and self-efficacy using the Perceived Stress Scale^18^ and Caregiver Inventory^19^ during 1 of 4 treatment or control visits.

#### Intervention Group

The data from the I-HOPE, demographic assessments, and assessment of functional abilities are used by the interventionist to develop an environmental modification intervention plan. Environmental modifications addressing basic ADLs are installed prior to discharge if possible. On returning home, the participant receives the remaining intervention visits, which focus on resumption of activities in the home and community. Additional environmental supports are provided as needed, and the occupational therapy interventionist and participant work together on poststroke community reintegration by using strategy-training techniques. Problem areas addressed are participant specific (ie, tailored), but all participants receive identical intervention components. The standardized components include assessment, identification of problematic activities (and environmental barriers), identification of solutions, implementation of solutions selected by the participant, training, and active practice of daily activities in the home and community.

#### AC Treatment

The control group experiences the same effects of time and attention in the home but no effect on the outcome of interest.^20^ A trained graduate assistant provides four 75-minute sessions. Topics include stroke symptoms, risk factors and preventing stroke recurrence, nutrition, managing emotions, sleep, fatigue, pain, social support, and sexuality. Environmental barriers are not addressed in the educational sessions.

#### Follow-up Period for All Participants

Participants with stroke and their caregivers are reassessed after intervention (T2) and at 6 and 12 months after stroke (T3 and T4). The follow-up activity, participation, and process assessments are conducted in the home. Falls and health care utilization are collected monthly by telephone.

### Data Collected

Assessments used to collect data for the primary, secondary, and exploratory outcomes are listed in the Table.^17,18,19,21,22,23,24,25,26^ All measures are assessed at T1, T2, T3, and T4.

**Table.** Outcome Assessments and Variables

Variable	Measure^a^
**Primary Outcome**	
Participation	RNLI,^21^ an 11-item questionnaire, quantifies participation (basic self-care, functional mobility, avocational and productive pursuits, and travel in the community)
**Secondary Outcomes**	
Daily activity performance	SIS ADL domain,^22^ a stroke-specific assessment of health-related quality of life, discriminates across 4 Rankin levels of stroke severity (*P* ≤ .01)^23^ and demonstrated a moderate (0.44) pre-post effect size between groups in our pilot
	I-HOPE^17^ evaluates the performance of older adults in the home, measuring limitations in daily activities, self-reported performance, and satisfaction with performance of problematic activities
Barriers in the environment	I-HOPE environment subscale^17^ measures the magnitude of environmental barriers that influence performance
**Exploratory End Points**	
Daily activity performance	BI^24^ assesses the ability of an individual to care for him or herself
Depression	GDS, short form,^25^ a 15-item screening tool, identifies depression in older adults
Health-related quality of life	PROMIS Physical and Mental Health Scales,^26^ a rigorously tested measurement tool, measures patient-reported outcomes that have a major impact on quality of life across a variety of chronic diseases
Caregiver burden and stress	CGI^19^ is a valid and reliable measure, consisting of 4 subscales: managing medical information (3 items), caring for the care recipient (7 items), caring for oneself (5 items), and managing difficult interactions and emotions (6 items)
	PSS,^18^ a stress assessment instrument, measures the degree to which situations in an individual’s life are considered stressful

Abbreviations: ADL, activities of daily living; BI, Barthel Index; CGI, Caregiver Inventory; GDS, Geriatric Depression Scale; I-HOPE, In-Home Occupational Therapy Performance Evaluation; PROMIS, Patient-Reported Outcome Measurement Information System; PSS, Perceived Stress Scale; RNLI, Reintegration Into Normal Living Index; SIS, Stroke Impact Scale.

^a^ All measures were collected at 4 points: baseline, immediately after intervention, 6 months after stroke, and 12 months after stroke.

### Statistical Analysis

#### Intention-to-Treat Analysis

 We will perform our analyses using an intention-to-treat paradigm. It is not possible for participants to switch conditions, as 2 groups of therapists are trained to provide either the intervention or control visits and rehabilitation staff are blind to group allocation. We will exclude the data of any individuals who drop out prior to randomization. We will perform exploratory data analysis looking for extreme or otherwise unusual values. Nonnormally distributed and heteroscedastic data will be transformed as necessary.

#### Baseline Analyses

We will use unpaired *t* tests and χ^2^ tests to compare baseline characteristics in the 2 groups for descriptive information. When statistical assumptions are not met, we may use Wilcoxon or Fisher exact tests.

#### Missing Data

We expect missing values in the outcome measures because of dropout, death, missed assessment, or nonresponse. Our main analysis, a linear mixed-effects model, accommodates missing values of outcome variables under a missing-at-random assumption.^27^ Assuming that missing data occur at random, inferences will be valid even if we have differential dropout by intervention arm. If the missing data mechanism is not ignorable (ie, missing not at random), then mixed-effects selection models or pattern-mixture models will be used.^28^

### Primary Study Objectives

#### Primary Study Outcome Analysis

All data will be analyzed using SAS version 9.4 (SAS Institute). The primary analysis (testing primary hypothesis) will be based on a linear mixed model using baseline and 12-month Reintegration to Normal Living Index (RNLI)^21^ scores, accounting for the relationship between a participant’s repeated measurements and time. The fixed-effect portion of the model will have the form Yit = β0 + β1 × 12 months + β2Group + β3Group × 12month, in which *Yit* is the RNLI score for participant *i* at baseline (time 0) and 12 months (time 1), and *Group* indicates study arm. In this model, the baseline RNLI is modeled as a dependent variable.^27^ For improved precision, the model will be adjusted for baseline covariates including race, sex, depression, and length of hospital stay if an imbalance in covariates between arms is observed in baseline analyses. In this model, *β0* is the mean RNLI score for the control arm at time 0, and *β1* is the change in the mean RNLI from baseline to time 1 for the control arm; *β2* is the mean RNLI score for the treatment arm at time 0, and *β3* is the change in mean RNLI from time 0 to time 1 for the treatment arm. The primary hypothesis is that the difference in the change in RNLI scores from time 0 to time 1 between arms will be tested by examining β3, which estimates the difference.

#### Secondary Study Outcome Analysis

For secondary analyses of the change in Stroke Impact Scale (SIS)^22^ score and I-HOPE score at 12 months, we will use the same approach as for the primary analysis because these 2 outcomes are also continuous. We have overall type I error control for testing the 12-month change in these 3 analyses at the design stage. The significance level for testing is *P* < .016, and all tests will be 2-tailed. In addition to comparing the 12-month change, we will extend the model by including scores immediately after intervention and at 6 months to see whether the difference in outcomes is achieved at those points. Depending on the form of the time variable in the model, we will use appropriate regression coefficients or a linear combination of the regression coefficients to determine the difference in change of these scores between arms at certain points. Interpretation of these results should be cautious because we have not controlled for the type I error in these analyses. Because it is possible that severity of functional impairment after stroke may affect response to treatment, we will analyze impact of functional impairment on response to treatment. We will examine functional impairment by group interaction to examine possible differential intervention effects of functional impairment on community participation and performance of daily activities.

### Secondary Study Objectives

#### Safety

To determine whether the intervention poses no greater risk than AC, we will examine the differences in number of falls and rehospitalizations between groups. The statistical models for count data will be used for analyses of these 2 outcomes. Using the number of falls as an example, we will fit a Poisson regression model (with overdispersion adjustment if necessary), in which a dummy variable for the intervention arm is used. The regression parameter estimate for this dummy variable is the log of rate ratio of falls for COMPASS intervention vs control arms, and the exponentiation of the regression parameter estimate is the rate ratio. Using the parameter estimate and its standard error, we can construct a 2-sided 95% CI for the rate ratio. We expect the confidence interval for rate ratio will include 1, indicating no significant difference in the fall rate between the 2 groups.

#### Process Analysis and Economic Evaluation

Acceptability and feasibility will be evaluated to aid in the interpretability of the trial; COMPASS will have high acceptability (80% retention), high fidelity by therapists (95% of elements and 90% of dosage delivered), low safety risk (no increased rate of falls or health care use compared with the AC group), and high adherence (80% of modifications in use) at 12 months. We will conduct between-group comparisons of process end points collected at each point (time to first fall, number of injurious falls, health care utilization rate, dosage delivered, and adherence rate) using unpaired *t* tests or χ^2^ tests. We will compare the characteristics of patients who complete the assigned intervention with the characteristics of patients who do not for differences in stroke severity and comorbidities. Descriptive statistics will be used for costs per participant and adherence.

### Sample Size Calculation

The study is designed to have 80% power to reject 3 null hypotheses of equal mean changes in the primary and secondary endpoints (RNLI, SIS, and I-HOPE) using a 2-sided, 2-sample, unequal-variance *t* test with overall type I error less than .05. Based on our preliminary study data,^29^ the 3 alternative mean (SD) changes in intervention and control populations are 15.3 (22.6) vs 1.3 (23.4), respectively, for RNLI, 15.7 (16.1) vs 5.6 (9.1), respectively, for the SIS ADL domain, and 62.1 (26.1) vs 46.2 (18.8), respectively, for the I-HOPE. With a 1:1 allocation ratio, 130 patients (65 in each group) are needed for the RNLI outcome, 84 are needed for the SIS outcome, and 100 are needed for I-HOPE outcome. We will enroll 180 patients to account for a 30% attrition rate. This magnitude of between-group difference is considered clinically meaningful based on prior relevant literature and is achievable based on our pilot study. The sample size calculation includes the correlation between baseline and follow-up measures and is based on analysis of change scores, which is equivalent in efficiency to the proposed analytic model. Based on the number of participants with stroke, we will enroll an equivalent number of caregivers.

### Safety Reporting

Because risk in the proposed study is considered minimal, the principal investigator is monitoring the study for adverse events, serious adverse events, and adherence to the protocol. The principal investigator will be responsible for reviewing study progress and outcomes including recruitment, data quality, safety, and efficacy.

### Adverse Events and Serious Adverse Events

All serious adverse events will be reported to the Washington University Human Research Protection Office using the Electronic Serious Adverse Event Reporting System. Reports will adhere to the following timeframes: (1) death, immediately; (2) life-threatening, within 7 calendar days; and (3) all other serious adverse events, within 15 calendar days.

### Handling and Storage of Data and Documents

Data are directly entered into a REDCap database, a secure, web-based application designed to support data capture for research studies.^13^ The REDCap servers are securely housed in an on-site, limited-access data center managed by the Division of Biostatistics at Washington University.

### Dissemination Policy

Study results will be submitted to peer-reviewed journals and presented at conferences on occupational therapy, stroke, aging, and public health. After publication, study participants will be informed of the results of the study.

## Discussion

Most stroke survivors report the inability to perform ADLs, decreased quality of life, and reduced community participation.^23,30,31^ Inpatient rehabilitation does not typically address the environmental barriers stroke survivors face when returning to the community.^29^ As a result, patients leave IR without the necessary skills to successfully return home. Emerging evidence, including our pilot study,^32^ demonstrates that it is possible to intervene during the transition from IR to home using compensatory approaches. However, it is unknown if an environmental modification intervention to reduce excess disability and improve community participation in the stroke population is effective. The findings of this phase 2b COMPASS trial will fill a critical gap in stroke rehabilitation evidence by providing important information about the long-term participation and environmental barriers of stroke survivors. If effective, this program will reduce disability in ADL performance and improve participation outcomes. We anticipate findings will resolve significant unmet need among stroke survivors with residual disability. At the conclusion of this study, we will understand the intervention’s efficacy, acceptance, safety issues, and optimal end points.

There are significant strengths of this study. First, COMPASS is designed for rapid translation. The intervention incorporates the elements of the Reach, Effectiveness, Adoption, Implementation, and Maintenance dissemination framework. The intervention was designed to facilitate rapid uptake in everyday practice, and it has standardized protocols for delivery with high fidelity. Second, patients at TRISL are enthusiastic about participating. Patient satisfaction surveys at TRISL indicate patient requests for home modifications and continued stroke education after discharge. Staff therapists and attending physicians at TRISL are excited about the study and are eager to make referrals to the study.

### Limitations

There are potential limitations to this study. This is a single-site design, which reduces the generalizability of the findings. Patients at TRISL are comparable nationally regarding length of stay and treatment; however, our pilot sample had a higher percentage of African American participants than in the general population of stroke survivors. It is also possible that baseline severity of disability may affect the response to the intervention. It may be that those with the most severe functional impairment derive the greatest benefit. Conversely, it is possible that the response to the intervention will be blunted in more impaired participants. We will conduct subanalyses to explore if a differential effect exists.

## Conclusions

We designed this randomized clinical trial to investigate the efficacy and safety of a novel enhanced rehabilitation transition program to reduce environmental barriers and improve daily activity performance and community participation. This study targets individuals 50 years and older who have experienced an acute stroke. Trial findings have the potential to provide evidence for the efficacy and safety of a transition program designed to increase the independence of stroke survivors. If this study finds that the novel enhanced rehabilitation transition program is successful, future research could be extended to other IR sites and populations in a phase 3 trial.

## Notes

Supplement. **Trial Protocol**
